# What constitutes a stigma? A review of isolated pores in raphid diatoms (Bacillariophyceae) and the value of precise terminology

**DOI:** 10.1111/jpy.13522

**Published:** 2024-11-17

**Authors:** Eileen J. Cox, Bart Van de Vijver

**Affiliations:** ^1^ The Natural History Museum London UK; ^2^ Meise Botanic Garden Research Department Meise Belgium; ^3^ Department of Biology – ECOSPHERE University of Antwerp Wilrijk Belgium

**Keywords:** buciniportula, cuniculus, fistula, isolated pores, SEM, stigma, stigmoid, terminology

## Abstract

Scanning electron microscopy has revealed variation in the ultrastructure of distinctive isolated pores in or near the central area of raphid diatoms, with different types of pores being restricted to phylogenetic groups. Thus, the widespread use of the term stigma for all such pores not only hides the structural diversity but also obscures the phylogenetic distribution of the different types. This paper provides images of the different types of isolated pores, particularly refining the discrimination of variants within the Cymbellales, and reveals some interesting ecological patterns. Revised definitions of stigmata and stigmoids are proposed, together with the recognition and definition of another type of stigmoid. The restricted distribution of more precisely defined pore types shows the importance of consistent use of terminology and its relevance to phylogenetic studies.

**Abbreviations**
LM: light microscopy
SEM: scanning electron microscopy

## INTRODUCTION

Distinctive isolated pores, of contrasting structure to the areolae composing the striae, occur near the central raphe endings in several raphid diatom genera. Van Heurck (1885, p. 64) noted the presence of a peculiar structure (“granules isolés”) in the central area of several *Cymbella* species such as *C. neocistula* (“2 à 5 granules isolés”) and *C. tumida* (“un ou deux granules isolés”). However, the first comment recognizing their significance seems to have been published by Cleve (1894), who introduced the term stigma for the isolated pores in five *Cymbella* species (*C. australica*, *C. tumida*, *C. janischii*, *C. mexicana*, and *C. punctifera*): “In one division of *Cymbella* there is in the middle of the central nodule or on its ventral side a peculiar punctum or pore, for which I use the name stigma” (Cleve, 1894, p. 157). But he (Cleve, 1894) discriminated this from one or more isolated pores at the ends of striae in some other *Cymbella* species such as *C. cymbiformis*, *C. neocistula*, *C. affinis*, *C. turgidula*, and *C. tumidula*. (“At the ventral side of the central nodule are two small puncta, ending the median striae” Cleve, 1894, p. 171.) Elsewhere, in his introduction to *Gomphonema* Ehrenb., Cleve (1894, p. 178) not only refers to the presence of “an isolated punctum or stigma” or a “unilateral row of stigmas” in many species (including *Gomphonema geminatum* = *Didymosphenia geminata*) but also uses the presence or absence of “stigmata” in his artificial key to *Gomphonema* species. Cleve (1894, p. 129, 130) had also noted the presence of an isolated pore on one side of the central nodule in *Navicula mutica* (= *Luticola mutica*), although this was structurally different to the isolated pores in the Cymbellales. Clearly, Cleve (1894) was limited in the discrimination of the finer structure of such pores, being solely dependent on nineteenth‐ in t;century light microscopy, and could only use number and position for comparison. But the term stigma was already being applied to what we now know are several contrasting structures within the cymbelloid and gomphonemoid diatoms.

With the introduction of scanning electron microscopy, it became clear that the ultrastructure of isolated pores and stigmata varied significantly between genera (Krammer, 1979, 1997a, 1997b; Appendix S1 in the Supporting Information). Krammer (1979) used the position of the pores in relation to the dorsiventrality of the valves (dorsal vs. ventral) as a discriminating feature, although (in both cases) ontogenetically, the pore position was on the primary side of the valve. Three years later, Krammer (1982) created the term *stigmoid* for isolated pores in the genera *Encyonema*, *Encyonopsis*, and *Gomphocymbella* that lacked the convoluted (cracked/fissured, according to Krammer) internal structure seen in *Cymbella* sensu stricto and opened at the end(s) of striae rather than separately. However, the internal openings of what he (Krammer, 1982) came to recognize as stigmata were also at the ends of striae, and as ontogenetically both ventral and dorsal sides could be the primary side of the valve, that particular criterion separating stigmata and stigmoids is called into question. In 2002, Krammer (2002, plates 4, 5) illustrated the variation in stigma (sensu Krammer) structure within cymbelloid taxa, both in position on the valve and internal structure. This showed that both stigmata and stigmoids (sensu Krammer) could open near the ends of central striae, although all his examples of stigmata (Krammer, 2002) were generally more complex internally (with rugged or serrated margins according to Krammer). It was also clear that these structures needed more careful discrimination.

Ross et al.'s (1979) earlier definition of a stigma had recognized that the internal structure and position of stigmata could differ, but they did not discriminate them any further. They also provided a definition of a cuniculus, another feature in which a distinct isolated pore is observed in the external central area of some *Parlibellus* species, opening internally at the ends of the raphe fissures (Cox, 1978, 1988). Later, Round et al. (1990) used stigma(ta) to refer to the isolated pore(s) in a broad array of genera: *Cymbella*, *Didymosphenia*, *Gomphoneis*, *Gomphonema*, *Gomphocymbella*, *Luticola*, and *Proschkinia*. They noted that *Encyonema* does not possess a true stigma and remained vague over the isolated pore in *Reimeria*, calling it “an isolated pore (stigma)” (Round et al., 1990, p. 486, 492, 494, 496, 498, 500, 532, 596), probably following Kociolek and Stoermer (1987) who had termed it a stigma.

Hustedt (1962) termed the isolated pore near the central nodule of several *Navicula* Bory species, now transferred to *Proschkinia*, either a stigma, an isolated pore, or a mucilage pore (“Gallertporus”), the latter later being observed to be structurally similar to the isolated pore in *Fistulifera*, termed *fistula* by Lange‐Bertalot (1997). More recently, Riaux‐Gobin and Compère (2009) described a new genus, *Olifantiella*, with an unusual internal structure that they termed a *buciniportula*, showing some similarity to the isolated pore of *Luticola*. A similar structure (simply called a stigma) had been observed in the earlier described genus, *Labellicula* (Van de Vijver et al., 2005), with the similarity between *Labellicula* and *Olifantiella* being later discussed by Van de Vijver et al. (2016). Both genera are currently recognized as taxonomically valid (Guiry & Guiry, 2024). The small valve dimensions of both genera made it initially difficult to study the ultrastructure of these isolated pores in detail, and it is only by using high‐resolution scanning electron microscopy (SEM) that it has become possible to begin to understand these ultrastructures in these genera in more detail and to consider them in a phylogenetic and ecological context.

Molecular phylogenetic analyses, based on one or more genes, are increasingly being applied to diatoms, including representatives of some of the above genera (Abarca et al., 2023; Glushchenko et al., 2022; Jahn et al., 2019; Kermarrec et al., 2011; Kezlya et al., 2021; Kim et al., 2020; Kulikovskiy & Kociolek, 2014; Majewska et al., 2019; Nakov et al., 2014; Tuji, 2020; Yana et al., 2022). These analyses allow us to see to what extent particular morphological features are distributed across clades and to infer their potential evolution. It is, however, clear that how we refer to these contrasting structures is relevant to their integration into phylogenetic analyses. If the same term is used to refer to significantly different structures, this potentially generates erroneous interpretations of relationships when working from published literature without going back to original illustrations every time (Cox, 2012). Does the terminology reflect homology, and are there any potential correlations with their physiology or ecology? This paper provides an overview of the morphological variation of isolated pores in raphid diatoms and recommends the use of terms that appropriately document that ultrastructural variation and reflect potential homologies. However, although it may be possible to infer the type of isolated pore in a particular specimen from its position on the valve and other morphological features allowing taxonomic identification, the presence of a particular pore type can only be confirmed by SEM study.

## MATERIALS AND METHODS

### Specimen preparation and microscopy

Subsamples from various materials containing species with isolated pores were prepared following the method described in van der Werff (1955). Small volumes of the samples were cleaned by adding 37% hydrogen peroxide and heating to 80°C for about 1 h. The reaction was completed by the addition of saturated potassium permanganate. Following digestion and centrifugation (three times for 10 min at 3700 rpm), the resulting cleaned material was diluted with distilled water to avoid excessive concentrations of diatom valves on the slides. Cleaned diatom material was mounted in Naphrax (refraction index 1.65) for light microscopy (LM). The resulting slides were analyzed using an Olympus BX53 microscope at 1000x magnification (UPlan FL N 100x objective, N.A. 1.30), equipped with differential interference contrast (Nomarski) optics and the Olympus UC30 Imaging System, connected to the cellSens Standard program. For scanning electron microscopy, part of the suspension was filtered through 5‐μm Isopore™ polycarbonate membrane filters (Merck Millipore), pieces of which were fixed on aluminum stubs after air‐drying, coated with a 20‐nm platinum layer, and studied using a ZEISS ULTRA field emission scanning electron microscope at 2 kV (Natural History Museum London, United Kingdom). Slides and stubs are archived in the BR‐collection (Meise Botanic Garden, Belgium). Plates were prepared using Adobe Photoshop CS5.

### Terminology with particular reference to the Cymbellales

The two sides of dorsiventral valves were traditionally discriminated by shape, with the wider, more convex side being designated dorsal and the usually narrower, less convex to concave side being designated ventral. Raphe paths, striae, positions of isolated pores, and orientations of cell contents (nucleus, pyrenoid) were also described in relation to this. Thus, the terminal raphe fissures of *Cymbella* are deflected toward the dorsal side, those of *Encyonema* to the ventral side. The nucleus of *Cymbella* is located against the ventral side, that of *Encyonema* against the dorsal side. However, ontogenetically, based on the position of the nucleus (against the primary side of the valve), the ventral side of *Cymbella* and dorsal side of *Encyonema* both represent the primary sides of the valves (Mann, 1981). In ostensibly bilaterally symmetrical valves, primary and secondary sides can be discriminated by the position of the Voigt discontinuity and the terminal fissure curvature of the raphe slits (Mann, 1981, 2006). We have therefore related isolated pore structures to the primary or secondary sides of the valve.

We also discriminated between Cleve's original use of stigma (stigma sensu stricto in *Didymosphenia*, *Oricymba*, and some *Cymbella* spp.) and its broader application by Krammer (1979, across other *Cymbella* spp.), adopting Krammer's (1982) term stigmoid for the structures in *Encyonema*, *Gomphonema*, *Gomphoneis*, *Kurtkrammeria*, *Afrocymbella*, *Gomphocymbellopsis*, *Reimeria*, *Placoneis*, and *Geissleria*, although this is discussed in more detail below. There has been a lack of consistency in the application of these terms by recent authors, with stigma often being used to refer to all the different types of isolated pores across all these genera, most likely because of the application of the word stigma by Round et al. (1990) for almost all the genera with isolated pores that were known in 1990 (*Cymbella*, *Didymosphenia*, *Gomphoneis*, *Gomphonema*, *Gomphocymbella*, *Luticola*, and *Proschkinia*; e.g., Abarca et al., 2020; Kociolek et al., 2018; Levkov et al., 2013; Liu et al., 2021; Reichardt, 2015; Stone et al., 2020; Zhang et al., 2020). However, the isolated pores in some *Gomphoneis* have more recently been referred to as stigmoids (Kociolek et al., 2013; You et al., 2013). It should also be noted that the current definitions of stigmata in glossaries of online diatom sites (www.diatoms.org, https://museumwales.ac.uk) are also rather broad and somewhat out of date.

The introduction of the terms fistula and buciniportula followed SEM studies leading to the recognition of *Fistulifera* and *Olifantiella* as new genera, which showed that the internal structure of their isolated pores contrasted with that of stigmata and stigmoids and warranted new terms (Lange‐Bertalot, 1997; Riaux‐Gobin & Compère, 2009).

## RESULTS

### Light microscopy

**FIGURE 1:**
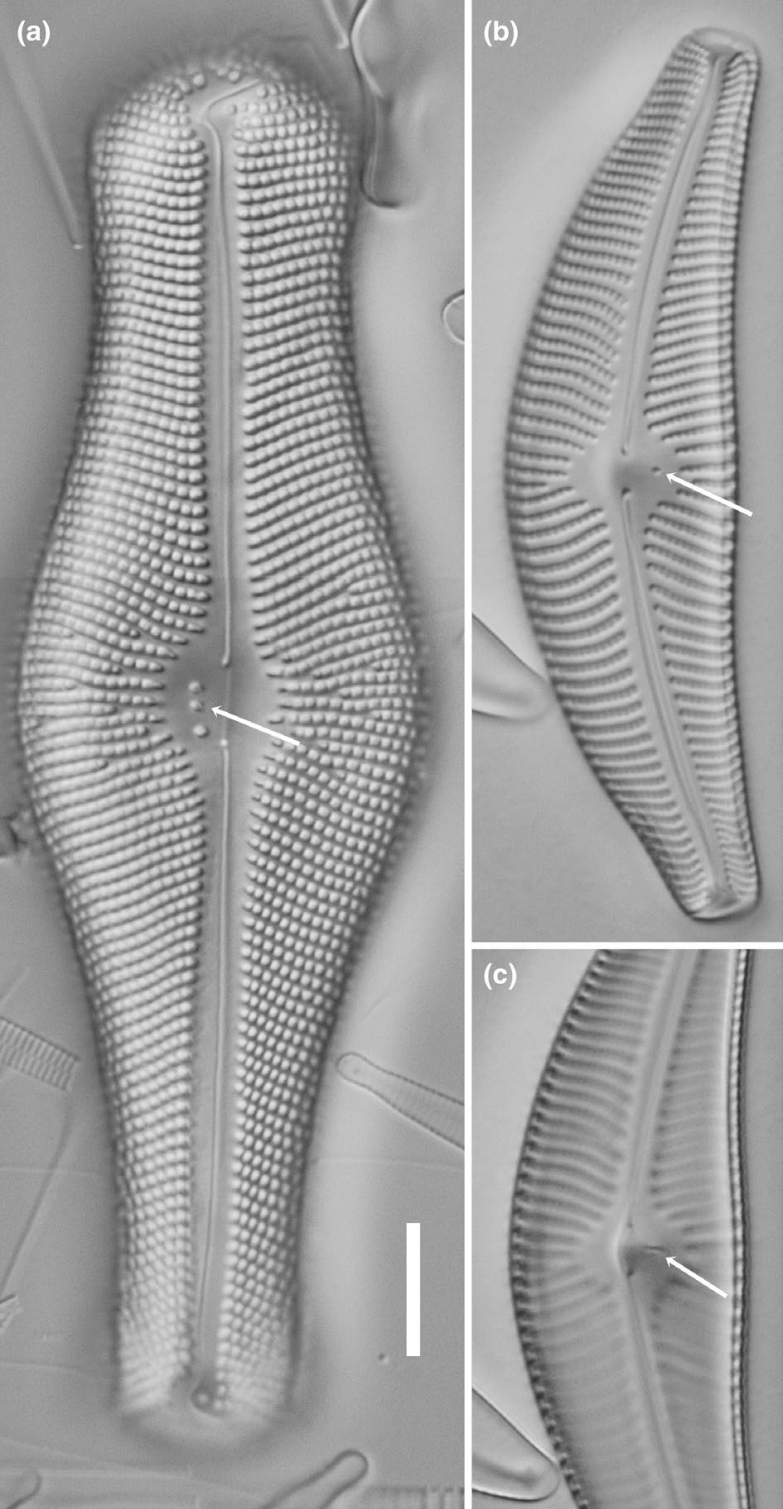
LM observations of taxa bearing a stigma. (a) *Didymosphenia geminata*. (b) *Cymbella tumida*, view of the valve exterior. (c) *Cymbella tumida*, view of the valve interior of the central area (different focal level). The arrows indicate the position of the stigma openings. Scale bar = 10 μm.

**FIGURE 2:**
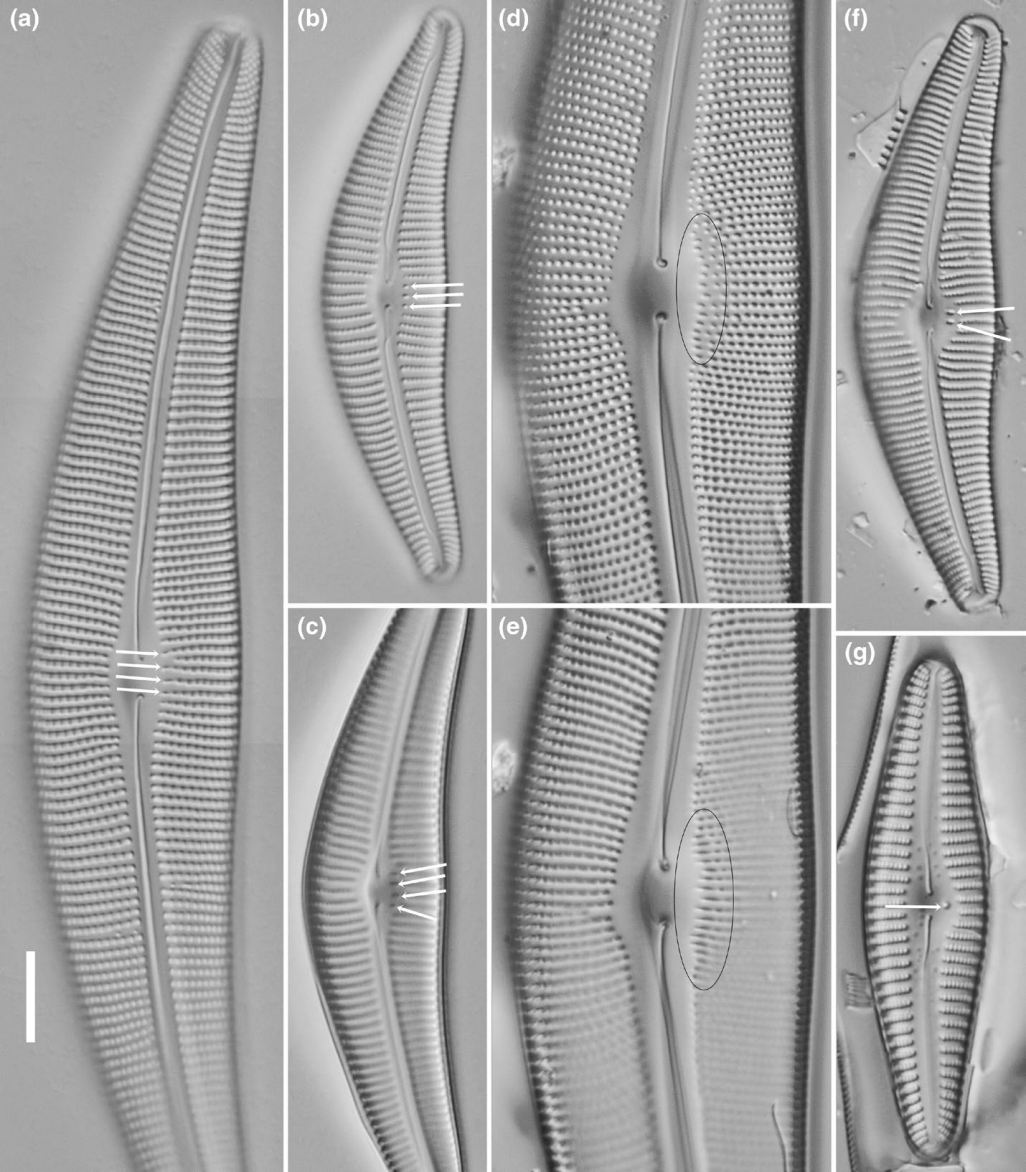
LM observations of taxa bearing a parastigma. (a) *Cymbella neolanceolata*. (b) *Cymbella neocistula*, view of the valve exterior. (c) *Cymbella neocistula*, view of the valve interior of the central area (different focal level). (d) *Cymbella aspera* view of the valve exterior. (e) *Cymbella aspera*, view of the valve interior of the central area (different focal level). (f) *Cymbella cymbiformis*. (g) *Oricymba* sp. The arrows indicate the position of the parastigma openings. The black circles on (e,f) show the position of the parastigmata. Scale bar = 10 μm.

The limits to resolution of light microscopy restrict the information it can provide to the number and position of any pores that appear different from those forming the striae. Thus, the stigmata of *Didymosphenia* and *Cymbella mexicana* are clearly located within or close to the central nodule (Figure 1), whereas in *Cymbella* sensu stricto and *Oricymba*, they lie at or very close to the central ends of the striae on the ventral (primary) side of the valve (Figure 2). In both cases, the number of pores can vary between species, for example, *Oricymba* (1), *D. geminata* (2–5), *C. mexicana* (1), *C. affinis* (1), *C. cymbiformis* (1–2), *C. turgidula* (2–3), *C. neocistula* (3–5), and *C. aspera* (7–10; Jüttner et al., 2010; Krammer, 2002; Metzeltin & Lange‐Bertalot, 2014).

**FIGURE 3:**
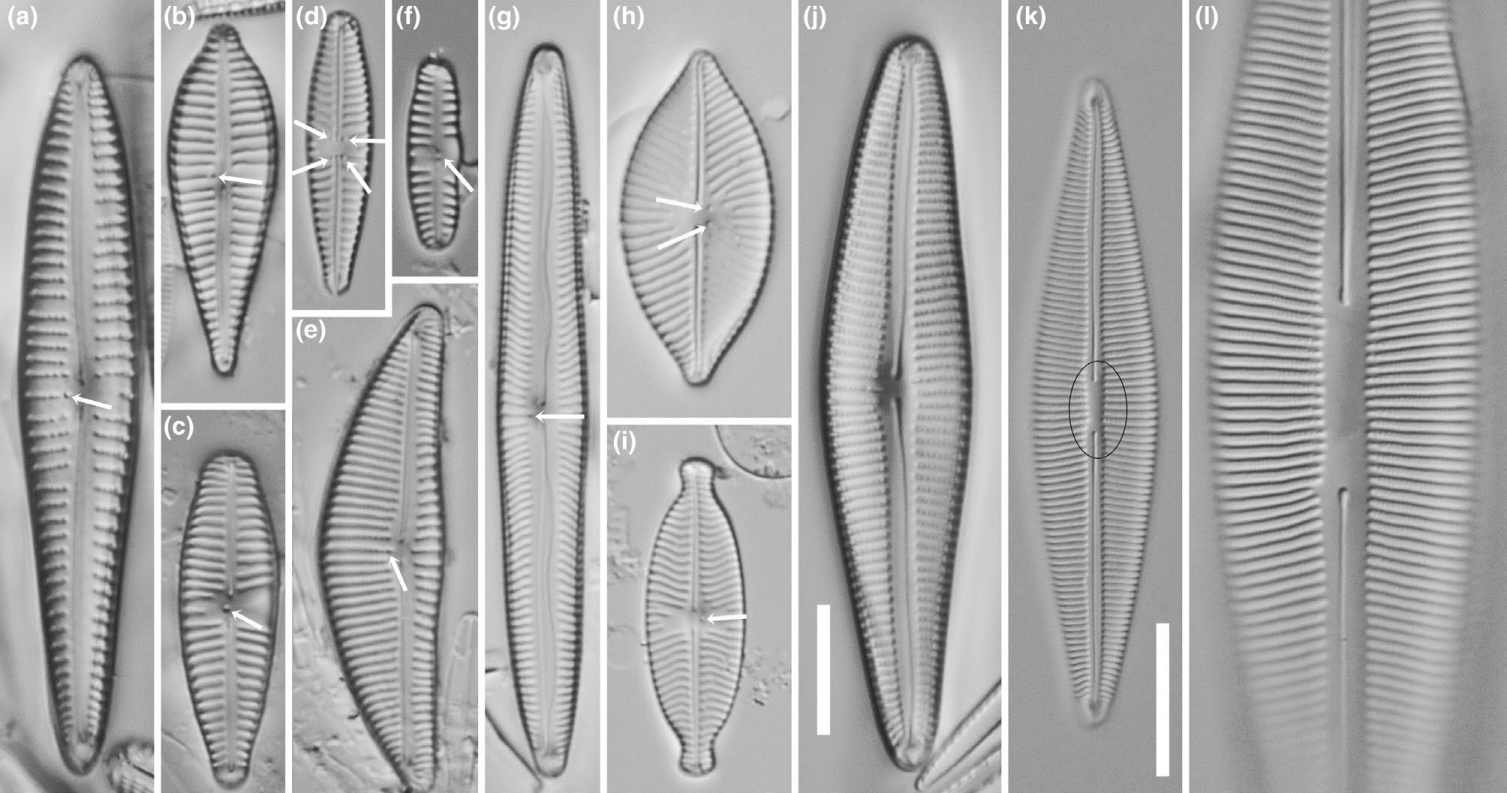
LM observations of taxa bearing a stigmoid. (a) *Gomphonema* sp. (b) *Gomphonema pseudoaugur* (c) *Gomphonema tergestinum*. (d) *Gomphonella* (e) *Encyonema*. (f) *Reimeria sinuata*. (g) *Kurtkrammeria neoamphioxys*. (h) *Placoneis clementispronina*. (i) *Geissleria decussis*. (j) *Afrocymbella barkeri*. (k) *Brebissonia lanceolata*. (l) *Brebissonia lanceolata*, detail of the central area. The arrows indicate the position of the stigmoid openings. The black circles on (k) show the position of the parastigmata. Scale bar = 10 μm except for (k) where scale bar = 20 μm.

When present, the isolated pores (stigmoids sensu Krammer) in *Encyonema*, *Kurtkrammeria*, *Gomphonema*, *Afrocymbella*, *Gomphocymbellopsis*, and *Reimeria* are usually distinctly different from the stria areolae and/or clearly separated from the central striae (Figure 3) on the primary side of the valve. However, in some (former) *Gomphonema* or *Gomphoneis* species, now *Gomphonella* (Jahn et al., 2019), four stigmoids could be seen, one each at the ends of four striae abutting the central area (Figure 3d). However, it should be noted that in their definition of *Gomphonella*, Jahn et al. (2019) indicated that stigmata or stigmoids were absent (aside from the occasional few isolated puncta in the central area, set aside from the striae, Jahn et al., 2019; Abarca et al., 2023), although Tuji (2020) has suggested that *Gomphoneis tetrastigmata* (with stigmoids) belongs in *Gomphonella*. When present, the isolated pores (stigmoids) in species of *Geissleria* and *Placoneis* were often paired and lay on the primary side of the central nodule, clearly separated from the central striae (Figure 3h,i). In *Brebissonia*, a change from biseriate striae to single areolae was just visible beside the central area under LM and allowed their recognition as potential stigmata or stigmoids (Figure 3k,l).

**FIGURE 4:**
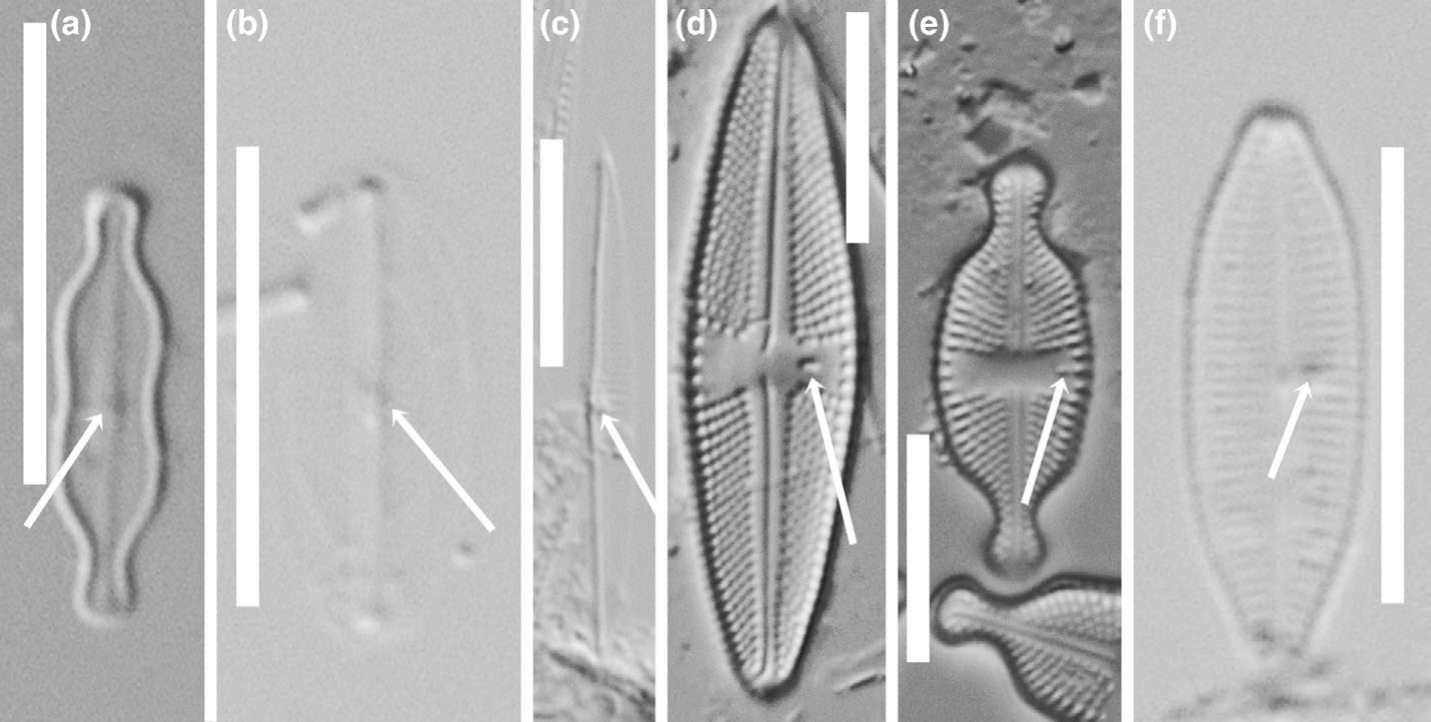
LM observations of taxa bearing a fistula (a–c) or a buciniportula (d–f). (a) *Labellicula subantarctica*. (b) *Fistulifera pelliculosa*. (c) *Proschkinia* sp. (d) *Luticola goeppertiana* (e) *Luticola ventricosa*. (f) *Olifantiella muscatinei*. The arrows indicate the position of the fistula (a–c) and buciniportula (d–f). Scale bars = 10 μm.

*Labellicula* did not have an obvious isolated pore when seen with light microscopy, but there was asymmetry in the refractiveness of the valve beside the central nodule (Figure 4a). Isolated pores in *Fistulifera* (Figure 4b) and *Proschkinia* (Figure 4c) were usually only seen as more or less refractive dots near the central raphe endings, whereas in *Luticola* (Figure 4d,e) and *Olifantiella* (Figure 4f), they were more obviously positioned between the striae and central area. The more recently described genus *Luticolopsis* (Levkov et al., 2013), showing structural similarities with *Labellicula*, also had an isolated pore between the central area and a short central stria (see Levkov et al., 2013, plate 202). As in members of the Cymbellales, the isolated pores were on the primary side of the valve in *Fistulifera, Luticola*, and *Proschkinia*; however, in *Karthickia* and *Olifantiella*, they were on the secondary side of the valve. It was harder to determine the ontogenetic position of isolated pores in *Labellicula* and *Luticolopsis* due to the difficulty in identifying the position of the Voigt discontinuity or curvature of the terminal raphe endings. In *Parlibellus*, the isolated pore was within the central nodule.

### Scanning electron microscopy

#### Stigmata sensu stricto

**FIGURE 5:**
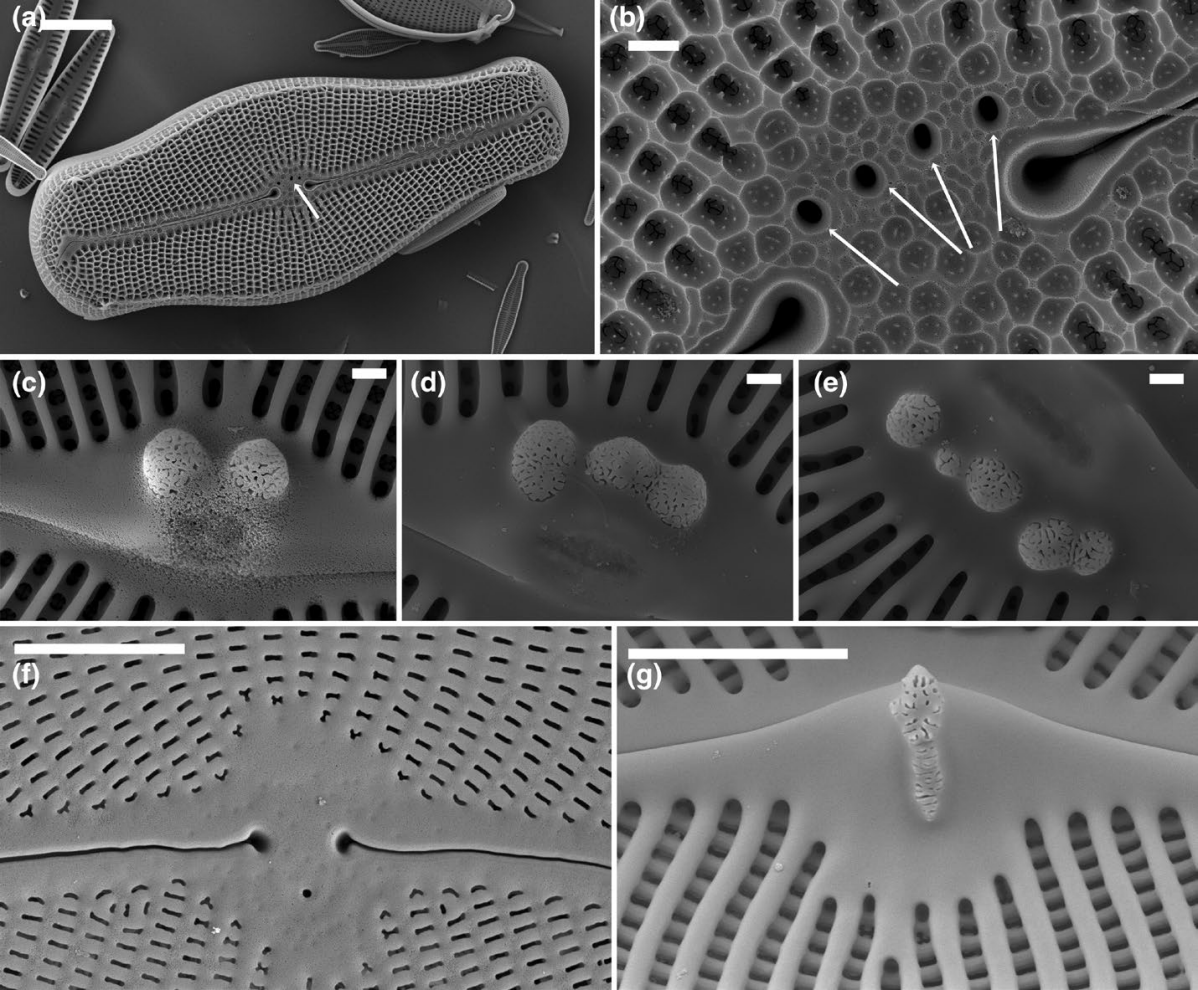
SEM observations of taxa bearing a stigma. (a) *Didymosphenia geminata*, external view of an entire valve. The arrow indicates the position of the stigmata. (b) *Didymosphenia geminata*, external view of the central area. The arrows indicate the external openings of the stigmata. (c–e) *Didymosphenia geminata*, three views of the stigmata in the valve interior indicating the variation in stigmata numbers. (f) *Cymbella tumida*, external detail of the central area with rounded opening of the stigma. (g) *Cymbella tumida*, internal detail of the stigma. Scale bar = 10 μm (a), 5 μm (f and g), 1 μm (b–e).

In external view under SEM, stigmata were seen as one or more unoccluded holes, within or to one side of the central area and the central raphe endings (Figure 5a,b,f). Internally, these opened as raised convoluted areas of silica on the primary side of the valve, often within the thickened central region of silica that overlay the internal central raphe endings, even if the external opening was more or less central within the central nodule (Figure 5c–e). With the use of a frustule cleavage method on *Cymbella janischii* (2021), Mayama and Mayama (2021) showed that that the convoluted internal surface was formed by radiating slits diverging from the simple external opening (figures 18, 21–23). The shape of the raised internal convoluted areas can be more or less circular in outline (*C. mexicana*, *Didymosphenia*: Figure 5c–e, and *C. janischii*: Mayama & Mayama, 2021, figure 18) or more elongated, as in, for example, *C. tumida*, extending onto the silica growth overlying the central raphe endings (Figure 5g).

#### Parastigmata (stigmata sensu Krammer 1979)

**FIGURE 6:**
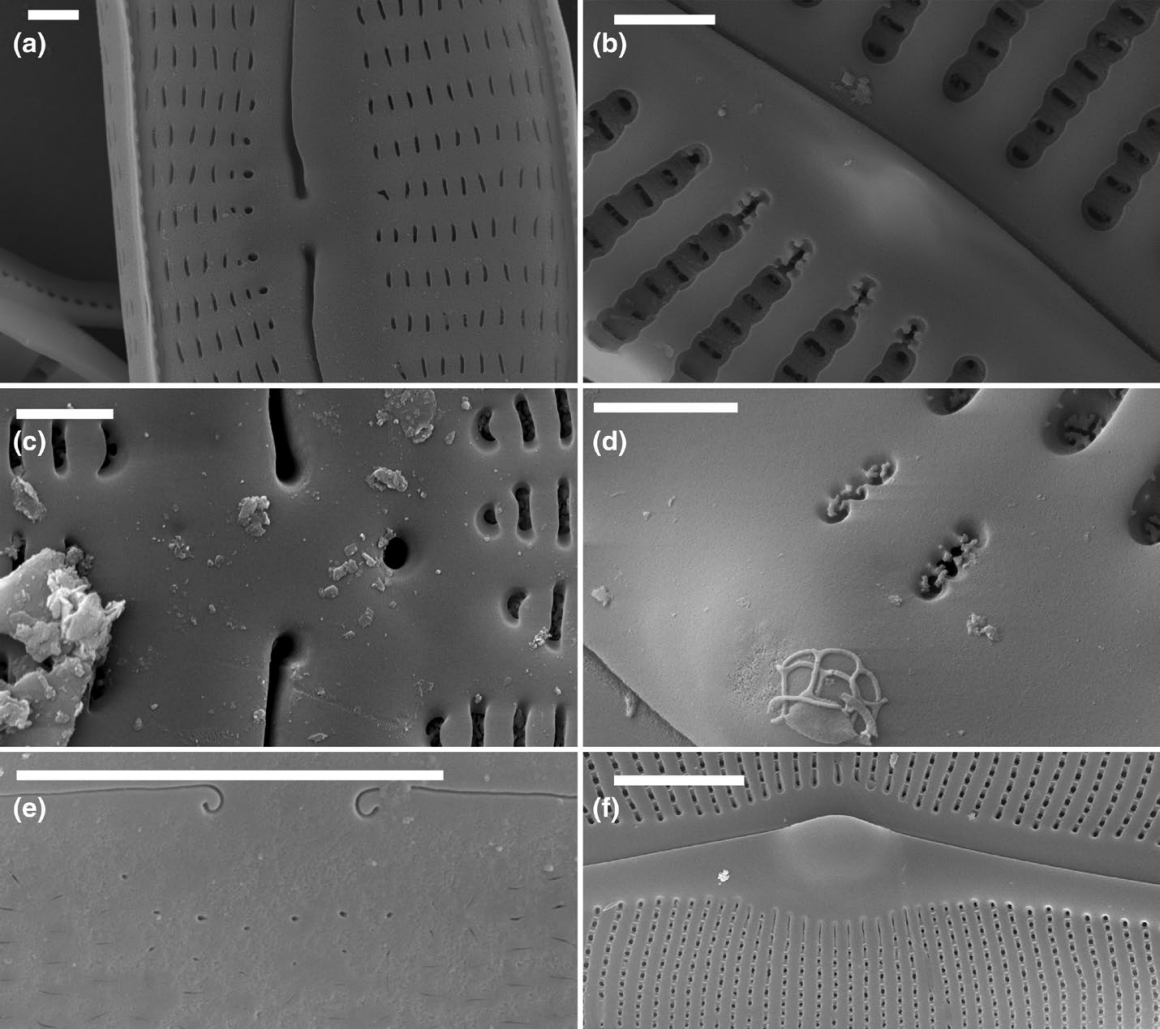
SEM observations of taxa bearing a parastigma. (a) *Cymbella neocistula*, external view of the central area showing the external openings of the parastigmata. (b) *Cymbella neocistula*, internal view of the central area showing the dentate openings of the parastigmata. (c) *Oricymba* sp., external detail of the central area with the large, rounded opening of the parastigma. (d) *Oricymba* sp., internal detail of the central area with the dentate openings of the parastigmata. (e) *Cymbella lanceolata*, external detail of the central area with the numerous very small, rounded openings of the parastigma. (f) *Cymbella aspera*, internal detail of the central area with the numerous slit‐like openings of the parastigmata. Scale bar = 5 μm (e), 1 μm (a–d, f).

Externally, the pore openings in this group were usually simple and rounded, on the ventral (primary) side of the valve (Figure 6a,c,e). Internally, two types of parastigma could be distinguished: those in which the internal openings had irregular or dentate margins (Figure 6b,d) and those where the internal openings were simply elongate with smooth edges (Figure 6f). Krammer (1979) related their positions to the dorsiventrality of the valve, that is, as ventral, which in these taxa is the primary side of the valve. In both cases, the external openings were to one side of the central area, and the pores opened internally at the proximal ends of the central striae. In species such as *Cymbella neocistula*, *C. cymbiformis*, and *C. neolanceolata* the internal openings had somewhat dentate margins (Figure 6b); in others, such as *C. aspera* (Figure 6f), the internal openings were more elongated than the usual areolae but had smooth margins. *Oricymba* species had internal openings with dentate margins (Figure 6d), as in the *C. neocistula* group (Jüttner et al., 2010; Krammer, 2002). Parastigmata with dentate margins were also present in some *Delicatophycus* species, for example, *D. williamsii* and *D. luiweii* (Liu et al., 2018, 2021).

#### Stigmoids—sensu Krammer

**FIGURE 7:**
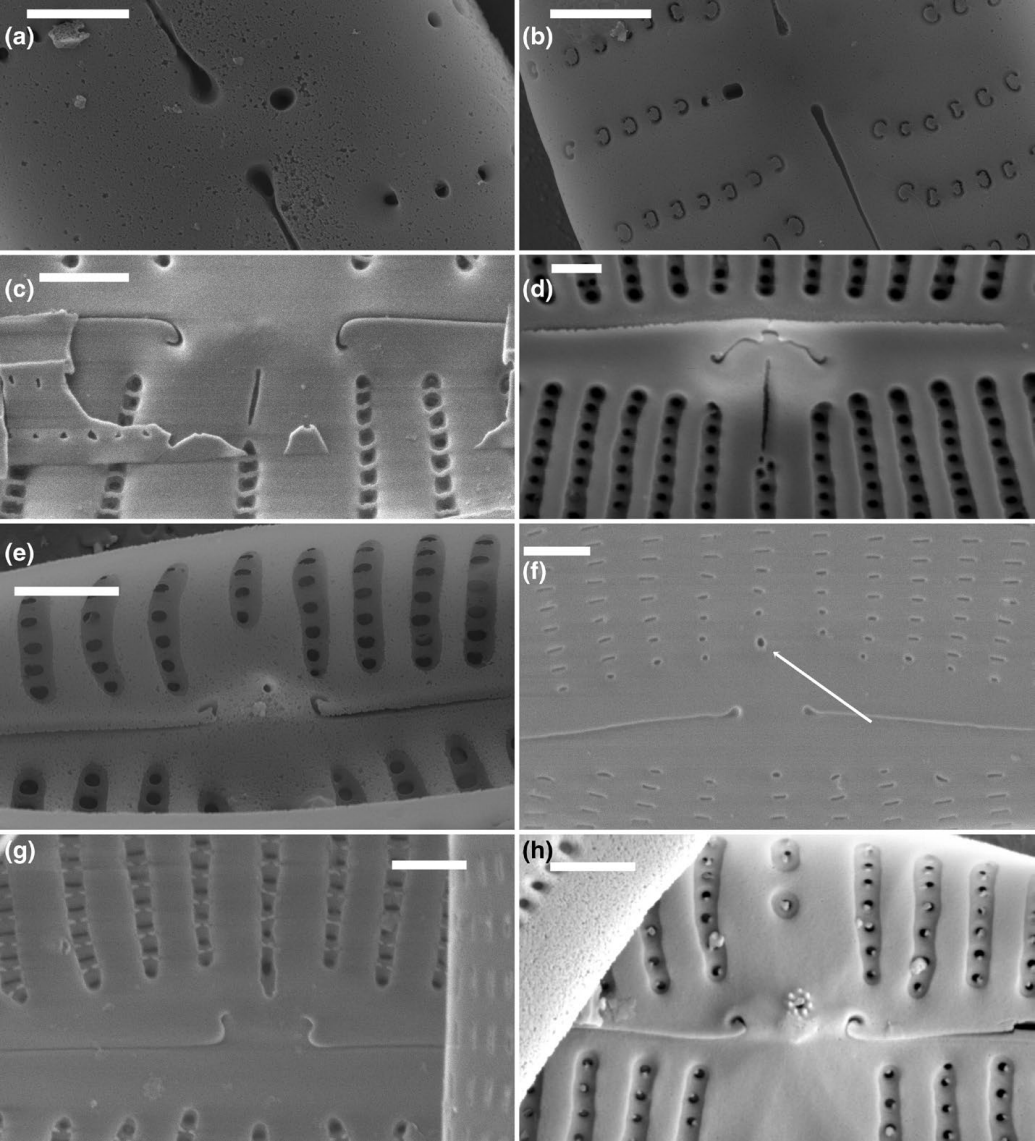
SEM observations of taxa bearing a stigmoid. (a) *Gomphonema* sp., external view of the central area showing the isolated external opening of the stigmoid, almost between the central raphe endings. (b) *Gomphonema* cf. *auritum*, external view of the central area showing the external opening of the stigmoid, at the end of a slightly shortened stria in the central area. (c) *Gomphonema* sp., internal detail of the central area with the slit‐like opening of the stigmoid. (d) *Afrocymbella barkeri*, internal detail of the central area with the slitlike opening of the stigmoid. (e) *Gomphonema* sp., internal detail of the central area showing the isolated internal opening of the stigmoid, almost between the central raphe endings. (f) *Encyonema silesiacum*, external detail of the central area with the rounded opening of the stigmoid (arrow). (g) *Gomphonema* cf. *pumilum*, internal detail of the central area showing the internal opening of the stigmoid, at the end of the middle stria. The stigmoid is located in a shallow depression. (h) *Gomphonema tergestinum*, internal detail of the central area with the more or less circular opening of the stigmoid, surrounded by small knobs of silica creating a flower‐like appearance. Scale bar = 1 μm.

**FIGURE 8:**
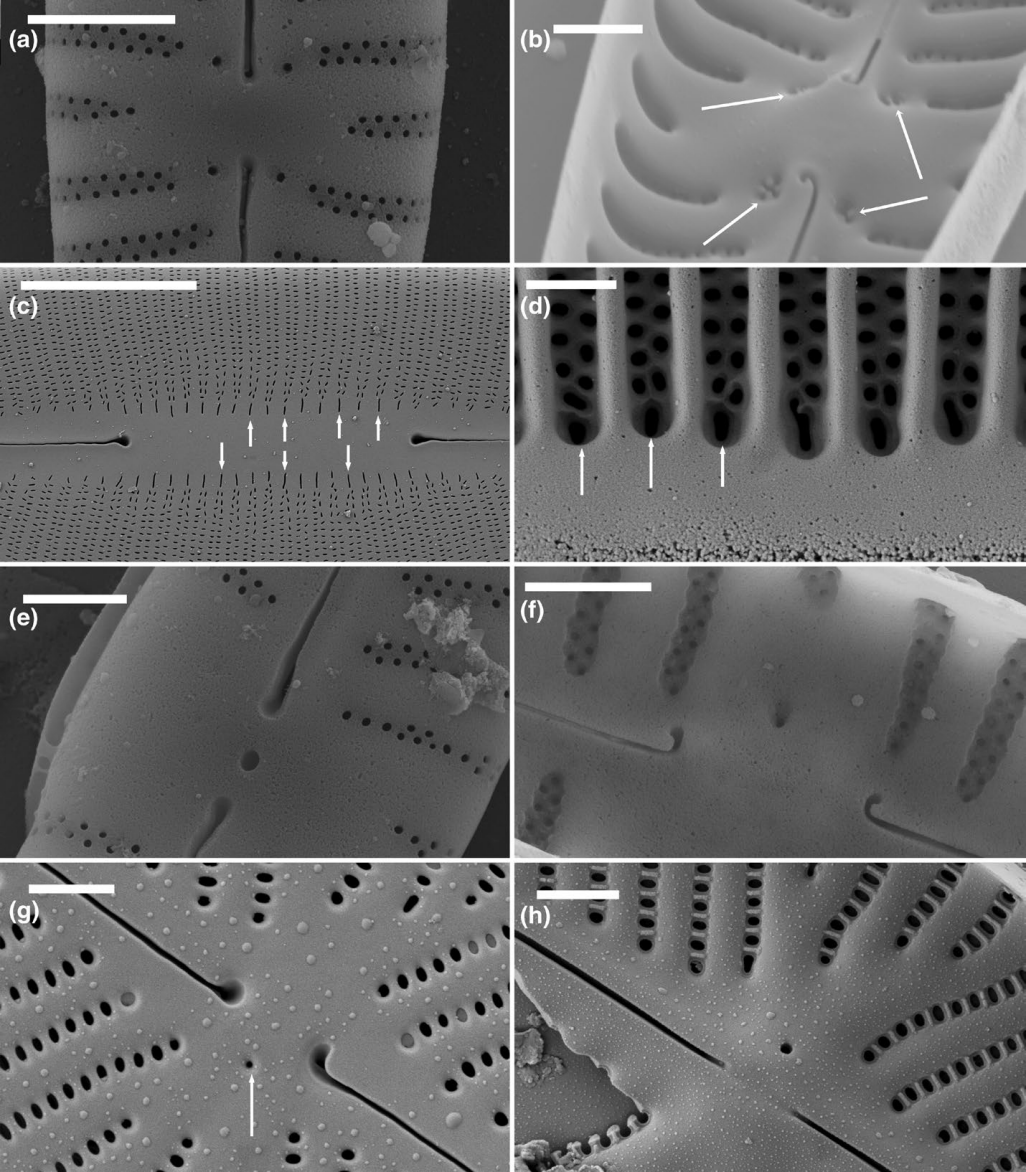
SEM observations of taxa bearing a stigmoid. (a) *Gomphonella* sp., external view of the central area showing the four small external openings of the stigmoids, at the end of the central striae (b) *Gomphonella* sp., internal view of the central area showing the four small internal openings of the stigmoids, indicated by the arrows (c) *Brebissonia lanceolata*, external detail of the central area with the slit‐like openings of the stigmoids (arrows) bordering the axial area. (d) *Brebissonia lanceolata*, internal detail of the central area showing some of the internal stigmoid openings (arrows). (e) *Reimeria sinuata*, external detail of the central area showing the isolated rounded external stigmoid opening, located between the central raphe endings. (f) *Reimeria sinuata*, internal detail of the central area showing the isolated rounded external stigmoid opening. (g) *Geissleria decussis*, external detail of the central area showing the small rounded stigmoid opening of the stigmoid (arrow), near the central raphe endings. (h) *Geissleria decussis*, internal detail of the central area showing the rounded stigmoid opening of the stigmoid. Scale bar = 1 μm.

Stigmoids, as defined by Krammer (1982), are simpler isolated pores, lacking the rounded, convoluted internal closure of stigmata, and they have their internal openings at the end of a central stria. However, more recent use of the term stigmoid has deviated from Krammer's original use with respect to the position of the internal opening, although all stigmoids lack any kind of convoluted or dentate siliceous ingrowths. Whereas stigmoid external openings were almost always round (Figures 7a,b,f and 8a,c,e,g), the internal openings varied in shape (Figures 7c–e, g, h and 8b,f,h): round in *Encyonema*, some *Gomphonema*, *Gomphonella* (according to Tuji, 2020), *Gomphocymbellopsis*, *Placoneis*, and *Geissleria* (Figure 8g,h); slit‐like in *Kurtkrammeria*, some *Gomphonema*, *Gomphadelpha* (although the authors refer to these as stigmata), and *Afrocymbella* (Figure 7d); and slightly elongate in *Reimeria* (Figure 8f). Single (occasionally two) stigmoids were observed on the primary side of the valve only, regardless of the dorsiventrality. External positions varied, not only sometimes clearly lateral to the central area but also closer to, or even between, the central raphe endings, for example, *Reimeria* (Figure 8e). However, internal openings were always more displaced toward the primary margin, suggesting a tubular connection between the exterior and interior. In *Afrocymbella*, *Kurtkrammeria*, *Gomphadelpha*, and most *Gomphonema* (but see below), the internal opening was usually clearly slit‐like and transapically orientated, varying in length between species (Figure 7c,d), although it could appear more rounded depending on the angle of view (Figure 7e). The slit‐like internal opening often lay midway between the laterally deflected, central raphe endings, but in others species, it terminated at one of the central striae. This resembles the situation in *Encyonema*, where a stigmoid could appear as a slightly more distinct, more rounded pore at the end of a central stria (Figure 7f), internally opening as a larger, more elongate pore terminating that stria (Figure 7g). The internal openings of a few *Gomphonema* spp., for example, *G. angustum*, *G. angustius*, *G. paratergestinum*, *G. supertergestinum*, and *G. tergestinum*, were more or less circular but surrounded by small knobs of silica creating a flower‐like appearance (Figure 7h).

Following the transfer of taxa in the *Gomphoneis tetrastigmata* species complex to *Gomphonella* (Tuji, 2020), some members of this genus are now considered to have four circular “stigmoids” at the central ends of biseriate striae close to both sides of the central raphe endings (Figure 8a,b). Internally, these may have irregular depressions or teeth‐like projections around their openings or are marked by one or a few transverse bars across the striae proximal to the central area (Figure 8b; Tuji, 2020, Figure 4a–d). However, the original description of *Gomphonella* (for species around *Gomphonema olivaceum*) stated that “there are no stigmoids or stigmata present on the valve face” (Jahn et al., 2019, p. 227), although there was a transition from biseriate to uniseriate striae in four striae approaching the central area, with the last areola in these striae often being slightly larger, more stigmoid‐like (Jahn et al., 2019, figures 5A, 8B, 8H, 9B, 9I, and 12G).

*Rexlowea* was separated from *Placoneis* in part because it did not have stigmoids (although not all *Placoneis* spp. have stigmoids), but the pores at the central ends of the striae around the central area are often larger and more distinct externally and elongated internally (Kociolek & Thomas, 2010, figures 14, 21). This is also the case for the rarely illustrated *Brebissonia lanceolata*, which possesses stigmoids on both sides of the central nodule (Mahoney & Reimer, 1986). In this taxon, the stigmoids open externally by transapical slits, perpendicular to the central area in contrast to the areolae in the biseriate parts of the striae, which are aligned parallel to the long axis of the valve (Round et al., 1990: 488 figure c, figure 8c). Internally, the stigmoids open as slightly elongate pores with smooth margins terminating the biseriate central striae alongside the central nodule (Round et al., 1990, p. 488 fig. g, figure 8d, arrows).

#### Stigmoids—*Karthickia*

Unlike the above taxa, the recently recognized cymbelloid genus *Karthickia* (Glushchenko et al., 2019) has a stigmoid on the secondary side of the valve with a more complex structure, showing a single external opening splitting to open internally at the ends of two striae (Yana et al., 2022, figures 20–22, 25, 27–31).

#### Fistulae

**FIGURE 9:**
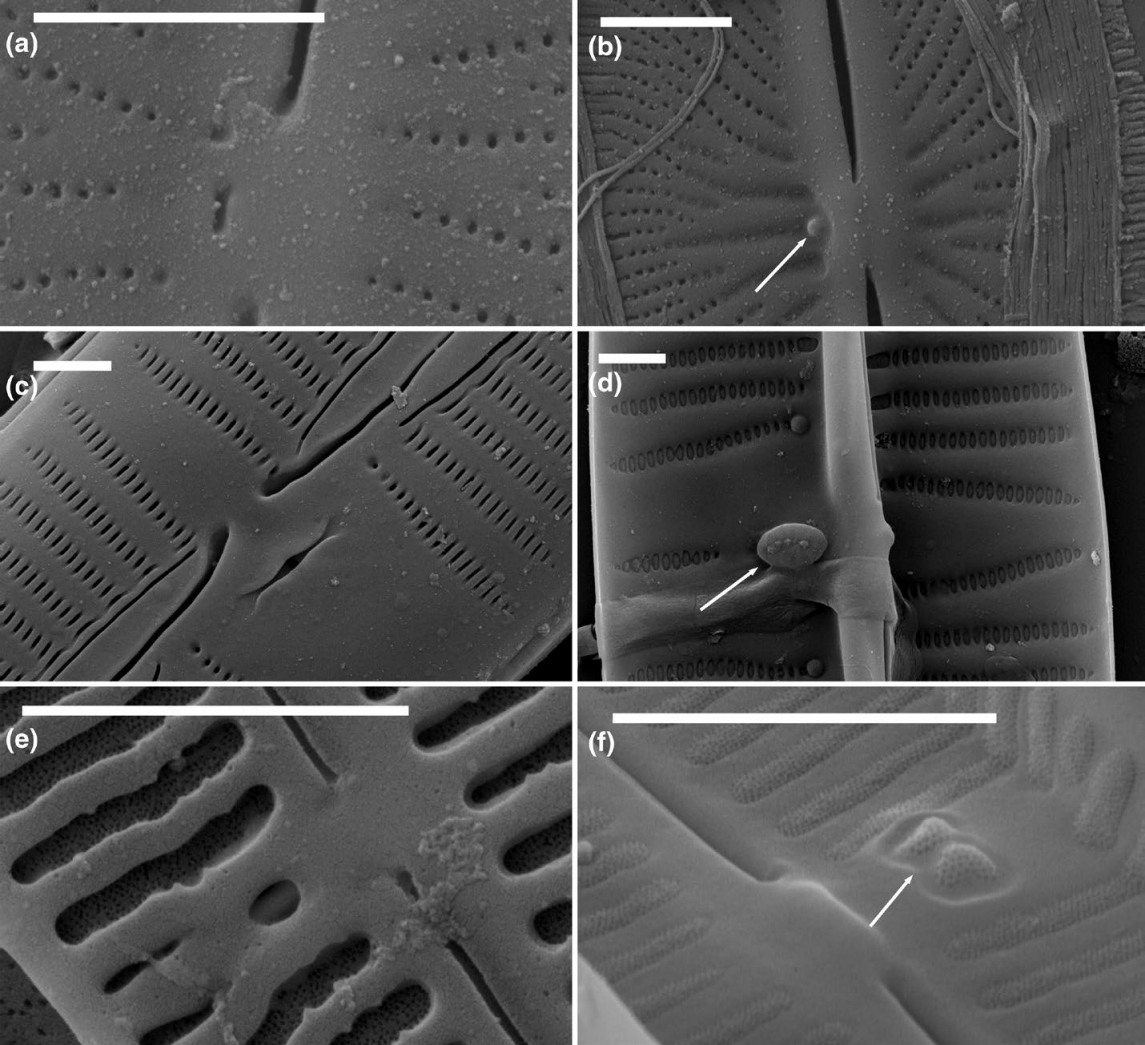
SEM observations of taxa bearing a fistula. (a) *Fistulifera pelliculosa*, external view of the central area showing the slit‐like opening of the fistula between the central raphe endings. (b) *Fistulifera pelliculosa*, internal view of the central area showing the fistula, presenting the raised, almost hemiglobular, hymenate internal closure. (c) *Proschkinia* sp., external detail of the central area with the large slit‐like opening of the fistula. (d) *Proschkinia* sp., internal detail of the central area with the knob‐like occlusion of the fistula (arrow). (e) *Labellicula subantarctica*, external detail of the central area with the large opening of the fistula. (f) *Labellicula subantarctica*, internal detail of the central area with the double hemiglobular coverings of the fistula. Scale bar = 1 μm.

Lange‐Bertalot (1997) defined a fistula based on the structure in *Fistulifera*, which has a simple, short slit externally (Figure 9a), with a raised, often almost hemiglobular, hymenate internal closure (Figure 9b). A similar structure was observed in *Proschkinia*, although the external opening in this genus was usually a longer slit, orientated along the apical axis and occasionally somewhat hidden by a ridge of silica. Internally, there may be one or a series of knob‐like, hymenate occlusions arranged perpendicular to the apical axis (Figure 9c,d). *Labellicula* has shown a similar structure, with internally two hemiglobular structures (Majewska et al., 2017, Van de Vijver et al., 2005; Figure 9e,f). Like the stigmata and stigmoids in the Cymbellales (except *Karthickia*), fistulae were observed on the primary side of the valve.

More recently, a similar structure with paired internal openings has been illustrated for *Luticolopsis vietnamica* (Levkov et al., 2013, plate 202: figures 22–25; plate 203: figures 5). This taxon has been considered to have a similar valve structure to *Luticola* (Levkov et al., 2013), but forms chains of cells, with the isolated pore displaced toward the margin of the valve, still opening to the exterior even when cells were linked as a chain. Its taxonomic position probably requires reassessment, possibly closer to *Labellicula* than *Luticola*.

#### Buciniportulae

**FIGURE 10:**
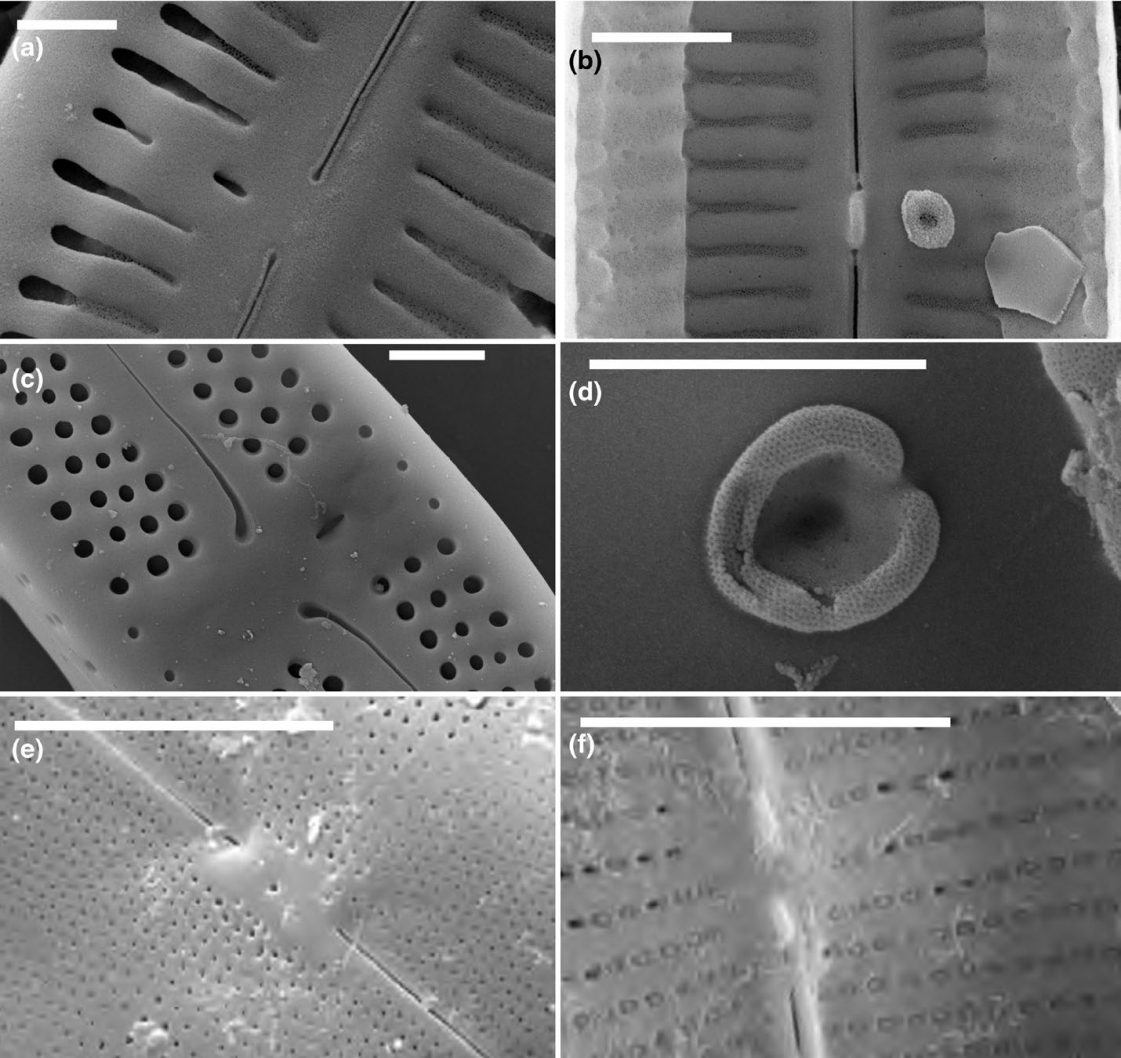
SEM observations of taxa bearing a buciniportula or a cuniculus. (a) *Olifantiella muscatinei*, external view of the central area showing the isolated slit‐like opening of the buciniportula. (b) *Olifantiella muscatinei*, internal view of the central area showing the trumpet‐like tubular buciniportula. (c) *Luticola denisei*, external detail of the central area with the large, isolated slit‐like opening of the buciniportula. (d) *Luticola denisei*, internal detail of the finely perforated occluded process of the buciniportula. (e) *Parlibellus* sp., external detail of the central area with the small opening of the cuniculus. (f) *Parlibellus* sp., internal detail of the central area with the longitudinal ridges at the end of the central raphe endings. Scale bar = 10 μm (e), 5 μm (f), and 1 μm (a–d).

Buciniportulae were first described in species of *Olifantiella* (Riaux‐Gobin & Compère, 2009) as structures with a simple external elongate opening connected to an internal tubular process with a flap‐like occlusion (Figure 10a,b). The length of the tubular process differed between species ranging from very long (see, for instance, Riaux‐Gobin & Al‐Handal, 2012, figures 8–10; Riaux‐Gobin & Compère, 2009, figure 18) to very short (Figure 10b). Other *Olifantiella* species have been described in which the internal opening is paired (e.g., *O. rodriguensis* Riaux‐Gobin & Al‐Handal, 2012, figures 20–21). Similarities have been drawn with *Luticola* (Riaux‐Gobin & Compère, 2009, p. 184) because it has, internally, a raised, lipped, flap‐like occlusion linked to a simple external opening, lacking, however, a protruding tubular structure (Figure 10c,d). As in *Olifantiella*, the flap‐like occlusion was very finely perforated, almost having a hymenate structure (Figure 10d). Mayama and Kobayasi (1986, figures 18–20, 44 and 45) described the internal occlusion as a “thin siliceous layer connected to the basal siliceous layer,” which represents exactly the same structure as in *Olifantiella* without the protruding tubular process (Mayama & Kobayasi, 1986, p. 175).

Although it can often be difficult to determine the primary and secondary sides of the valves in *Olifantiella* and *Luticola*, buciniportulae seem to be located on the secondary side of the valves, unlike stigmata and stigmoids, always present on the primary valve side.

#### Cuniculus

The term cuniculus was applied to the structure observed at the center of two *Parlibellus* species, *P. delognei* and *P. rhombicus*, which were illustrated by Cox (1978: figure 8–10, 16–22; 1988: figures 34, 36, and 38). This structure appeared as a simple external pore (occasionally two pores) at the center of the valve between the central raphe endings (Figure 10e) but had longitudinal perforated (hymenate) ridges terminating the raphe endings internally (Figure 10f; Cox, 1978: figures 8–10, 1988: figure 36).

## DISCUSSION

**FIGURE 11:**
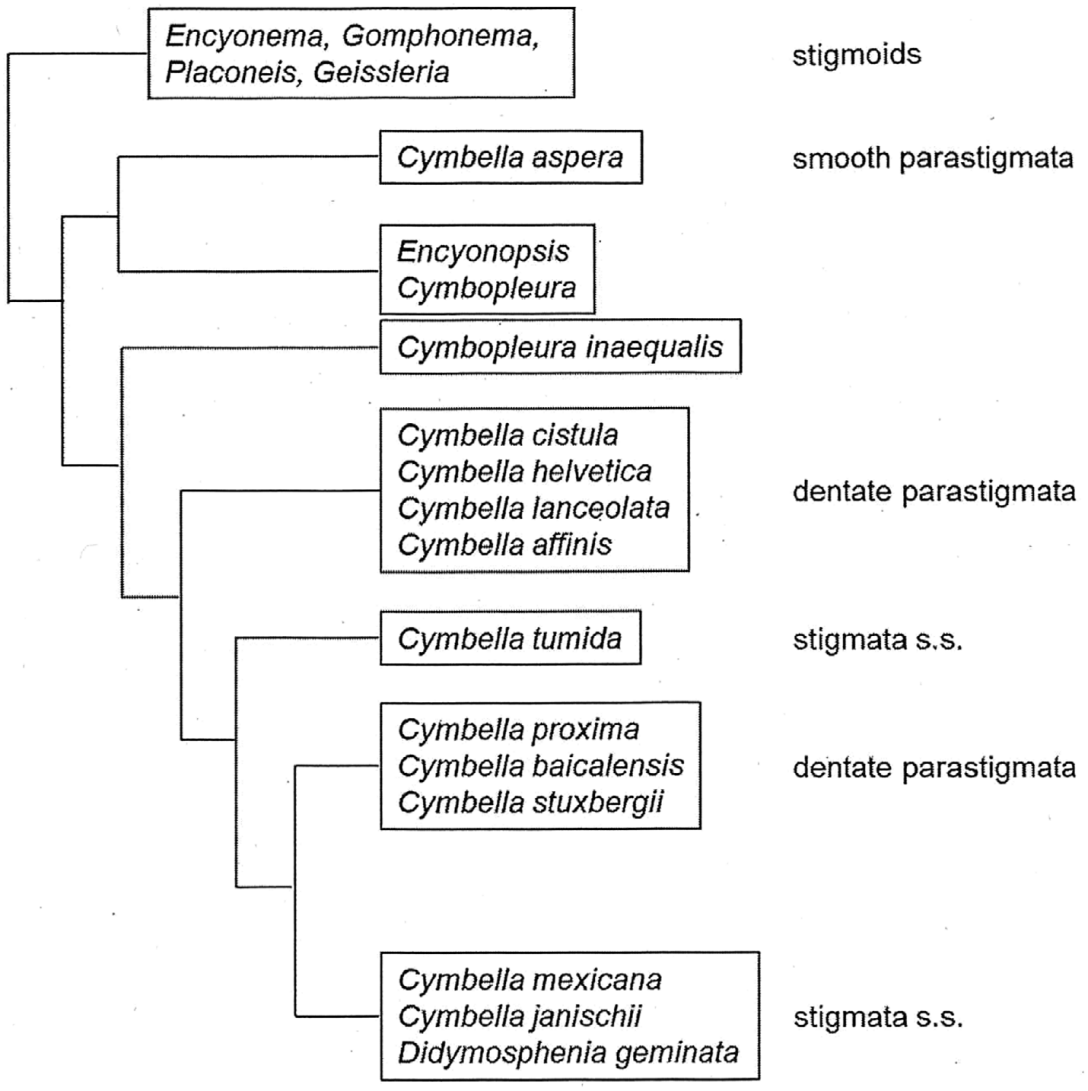
Diagram showing the distribution of different types of stigmata across *Cymbella* and *Didymosphenia* based on the maximum likelihood tree by Nakov et al. (2014, figure S1; adapted with permission).

It is clear that terminology around isolated pores, particularly for species within the Cymbellales has been confused (Cox, 2012), with some authors using stigma(ta) regardless of any variation in structure (Abarca et al., 2023; Van de Vijver et al., 2005), whereas other authors recognize differences in structure and use stigmoid, at least for some genera (Jahn et al., 2019). This, of course, assumes that ultrastructural details have been determined, and we suggest that if specimens have only been observed with LM the neutral term “isolated pore” should be used. Although some homology between the different types of isolated pores has been assumed, molecular analyses that have included cymbelloid taxa (Abarca et al., 2023; Glushchenko et al., 2022; Jahn et al., 2019; Kermarrec et al., 2011; Kezlya et al., 2021; Kulikovskiy & Kociolek, 2014; Nakov et al., 2014; Tuji, 2020; Yana et al., 2022) have revealed that different stigmata and stigmoid types fall in different clades (Figure 11 is based on a tree in Nakov et al., 2014 because this most clearly shows the relationship between several cymbelloid taxa and isolated pore structure. Later trees—using the same sequences—do not conflict with this).

Thus, *Didymosphenia*, *Cymbella janischii*, and *C. mexicana* with stigmata sensu stricto consistently fall in the same clade, sometimes with *C. proxima* and *C. stuxbergii* (with dentate stigmata sensu Krammer) as a sister clade between *Didymosphenia* + *C. janischii* + *C. mexicana* and *C. tumida* (Nakov et al., 2014, figures 3–5, S1). Other taxa with dentate stigmata sensu Krammer, for example, *C. affinis*, *C. neocistula*, *C. cymbiformis*, *C. helvetica*, and *C. neolanceolata* group together but separate from a clade with smooth stigmata sensu Krammer, for example, *C. aspera* and *C. baikalaspera*, which lie closer to *Cymbopleura* spp. (Nakov et al., 2014, figures 3–5, S1), which do not possess stigmata or stigmoids. We, therefore, suggest that these three types are different and propose that *stigma* be restricted for the isolated pores in *Didymosphenia*, *C. janischii, C. mexicana*, and *C. tumida*, in which the internal openings have raised, highly convoluted coverings. We propose a new term, *parastigma* (=stigma sensu Krammer), for the pores in most *Cymbella* species, with a relevant preceding descriptor. Thus, where the internal openings have teeth‐like projections, we call these dentate parastigmata; where the internal openings do not have these projections, we call these smooth parastigmata. *Oricymba* spp., for instance, possess dentate parastigmata using this terminology. It should also be noted that *Oricymba* is unusual in that there were two internal parastigma openings but a single simple external one. We presume that there is bifurcation of the connecting external–internal tube, but this requires confirmation.

A potential variant “stigma” has been shown in the recently established genus *Gomphosinica*, in which the internal opening formed a perforated mound rather than having a more convoluted surface (Kociolek et al., 2015, figures 30, 45, 61. 82, 99, 110, 131). Although Kociolek et al. (2015, figure 133) presented an “evolutionary scenario of phylogenetic relationships of freshwater gomphonemoid diatoms” (p. 178), this genus has not been included in any molecular analyses, and its “stigma” may not be homologous with a stigma sensu stricto. Unlike all other taxa with stigmata or parastigmata, *Gomphosinica* does not have hidden internal central raphe endings and may be a member of the Gomphonemataceae rather than the Cymbellaceae in which true stigmata are seen. All *Cymbella* and *Didymosphenia* taxa are separated from *Encyonema*, *Gomphonema*, *Reimeriai*, *Placoneis*, and *Geissleria* spp. with isolated pores (when present) that would be considered stigmoids (Glushchenko et al., 2022; Jahn et al., 2019; Kermarrec et al., 2011; Kezlya et al., 2021; Kulikovskiy & Kociolek, 2014; Nakov et al., 2014; Yana et al., 2022), while *Gomphonella* species form a clade of their own (Jahn et al., 2019; Tuji, 2020; Yana et al., 2022), separated from *Gomphonema*, *Encyonema*, *Reimeria*, *Placoneis*, *and Geissleria*. When stigmoids are present in *Placoneis* and *Geissleria*, they open internally as simple round pores, immediately below their external openings, whereas in *Encyonema*, *Gomphonema* and *Reimeria*, the internal stigmoid openings can vary from almost round to elliptical to slit‐like, with some taxa having characteristically long internal slits, for example. *Afrocymbella* and *Afrocymbella* have not been included in any molecular phylogenies to date, so their phylogenetic relationships to other stigmoid‐bearing taxa remain unknown. *Reimeria* forms a sister clade to some *Gomphonema* species. Although external stigmoid openings in *Gomphonema* are usually lateral (primary side) to the central raphe endings, they open almost centrally in *Reimeria*, between the raphe endings, although the internal openings are more lateral on the primary side.

Although the distribution of different stigma, parastigma, and stigmoid types across taxa in the phylogenetic trees generally matches the clades including those taxa, some *Cymbella* spp. lack parastigmata, while stigmoids are not invariably present in all *Encyonema*, *Placoneis*, and *Geissleria* species. Additionally, whereas, as far as we know, all the above stigmata and stigmoids always have a one‐to‐one relationship between their external and internal openings, the parastigma in *Oricymba* and the dichostigmoid in *Karthickia* have single external openings connected to two internal ones, that is, the connecting “tube” bifurcates. More detail on the 3D structure of the parastigmata in *Oricymba* and the ontogeny of both genera would be invaluable for determining any homology between them.

There is a major structural difference between the isolated pore structures in the Cymbellales, which are never fully occluded, and the fistulae, buciniportulae, and cuniculi present in some members of the Naviculales, which have hymenate closures. Although the phylogenetic relationships of *Fistulifera* and *Proschkinia* have been explored in a few publications (Kim et al., 2020; Majewska et al., 2019; Tseplik et al., 2022; Zgrundo et al., 2013), there are no molecular studies focusing on *Olifantiella* or *Labellicula*, and *Luticola* has rarely been included (but see Han et al., 2018, Kezlya et al., 2021). However, based on partial 18S rRNA gene sequences, a putative *Olifantiella* species grouped with *Luticola* (Han et al., 2018), distant from *Gomphonema*, which had been used as the outgroup. This could support the identification of the isolated pores of both *Olifantiella* and *Luticola* as buciniportulae. Similarly, a phylogeny based on partial *rbc*L and SSU rDNA gene sequences placed *Parlibellus* in the Stauroneidaceae, also within the Naviculales and remote from the Cymbellales (Kulikovskiy et al., 2019).

Despite the morphological differences, on which their taxonomic affiliations are based, fistulae and buciniportulae occur in taxa from marine or high conductivity waters, unlike the freshwater members of the Cymbellales. The internal closure of a cuniculus in *Parlibellus*, a marine genus, is also finely perforate, like a hymen. The contrast in closure may be linked to their ecology: hymenate occlusions occurring in taxa located in marine and high conductivity environments and possibly being important in osmoregulation in more saline waters.

### Revised definitions of terms

#### Stigma (=stigma sensu Cleve)

An isolated pore that opens externally near or within the central area, internally with a rounded or elongate, domed crenulate surface. Observed in *Didymosphenia* and some *Cymbella* spp., including *C. mexicana*, *C. janischii*, and *C. tumida*. The pores lie on the primary side of the valve or, if they are central, open internally on the primary side of the valve, within the silica flange that overlies the internal raphe endings.

#### Parastigma (=stigma sensu Krammer)

A distinctive pore at or near the end of one or several central striae, on the primary side of the valve, larger and more rounded than the stria areolae, more elongate internally. The internal openings may be smooth (smooth parastigma) or with more convoluted, dentate margins (dentate parastigma). Observed in *Cymbella*, *Oricymba* and some *Delicatophycus* spp., for example, *D. williamsii* and *D. liuweii*. *Cymbella* spp., with those with smooth or dentate parastigmata usually falling in different parts of published phylogenetic trees.

#### Stigmoid

A distinctive pore, usually at least slightly removed from the central end of a stria on the primary side of the valve, with a simple round external opening, but varying in shape internally, although never occluded or partially occluded. Internal openings may be round to elongate or slit‐like, sometimes curved. Some may be surrounded by small silica knobs, creating a flower‐like appearance, for example, *Gomphonema tergestinum*. The presence of four stigmoids, one at each end of the central striae flanking the central area have been observed in *Gomphonella* and some *Gomphonema* spp., such as the *G. tetrastigmata* group. The distinctive pores flanking both sides of the central nodule in *Brebissonia* are stigmoids.

#### Dichostigmoid

A dichostigmoid has only been observed in *Karthickia*, differing from stigmoids in being positioned on the secondary side of the valve, with a simple round external opening internally into two pores at the ends of central striae.

#### Buciniportula

A distinctive isolated pore, opening externally as an elongate pore, to one side of the central area, internally as one or two, often raised, tubular structures, with thin siliceous, finely perforated, flap‐like coverings. The height of the internal tube varies between species, particularly in *Olifantiella*, in which examples in with double internal openings have occurred. In *Luticola*, the internal structure is shorter, and only single internal openings have been observed. Although it is often difficult to identify unequivocally primary and secondary sides of the valve in these genera, buciniportulae seem to lie on the secondary sides.

#### Fistula

An isolated pore with a slit‐like external opening, aligned along the long axis of the valve, opening internally as one or more raised, spherical hymenate areas. The fistula lies on the primary side of the valve. It has been observed in *Fistulifera*, *Proschkinia*, and *Labellifera*.

#### Cuniculus

This structure appears as a simple external pore (occasionally two pores) at the center of the valve between the central raphe endings, but as raised, longitudinal perforated (hymenate) ridges terminating the raphe endings internally.

## AUTHOR CONTRIBUTIONS

**Eileen J. Cox:** Conceptualization (equal); investigation (equal); methodology (equal); writing – original draft (lead); writing – review and editing (equal). **Bart Van de Vijver:** Conceptualization (equal); investigation (equal); methodology (equal); writing – original draft (supporting); writing – review and editing (equal).

## Supporting information


**Appendix S1.** List of published images of different types of isolated pores.
